# Patterns and Possible Roles of LINE-1 Methylation Changes in Smoke-Exposed Epithelia

**DOI:** 10.1371/journal.pone.0045292

**Published:** 2012-09-18

**Authors:** Siriporn Wangsri, Keskanya Subbalekha, Nakarin Kitkumthorn, Apiwat Mutirangura

**Affiliations:** 1 Department of Oral and Maxillofacial Surgery, Faculty of Dentistry, Chulalongkorn University, Bangkok, Thailand; 2 Department of Oral and Maxillofacial Pathology, Faculty of Dentistry, Mahidol University, Bangkok, Thailand; 3 Department of Anatomy, Faculty of Medicine, Center of Excellence in Molecular Genetics of Cancer and Human Diseases, Chulalongkorn University, Bangkok, Thailand; Geisel School of Medicine at Dartmouth, United States of America

## Abstract

Tobacco smoking and reduced methylation of long interspersed element-1 (LINE-1) are crucial in oral carcinogenesis. 5′UTR of human LINE-1 sequence contains several CpG dinucleotides which are methylated in various proportions (0–100%). Methylation levels of many LINE-1s in cancer were reduced, hypomethylated. The hypomethylation of each LINE-1 locus can promote instability of genome and repress expression of a gene located on that same chromosome. This study investigated if cigarette smoking influences LINE-1 methylation of oral mucosal cells. The methylation of human LINE-1 in clinically normal oral mucosa of current smokers was compared to non-smokers. By using the combined bisulphite restriction analysis, each LINE-1 sequence was categorised into 4 patterns depending on the methylation status and location of the two 18-bp successive CpG from 5′ to 3′ including ^m^C^m^C, ^u^C^u^C, ^m^C^u^C and ^u^C^m^C. Of these, ^m^C and ^u^C represent methylated and unmethylated CpG, respectively. The DNA bisulphite sequence demonstrated that most CpGs of ^m^C^m^C and ^u^C^u^C were methylated and unmethylated, respectively. Nevertheless, some CpGs of each ^m^C^u^C or ^u^C^m^C allele were methylated. Imaging of the digestion products was used to generate %methylation value. No significant difference in the overall LINE-1 methylation level but the differences in percentages of some methylation patterns were discovered. The %^m^C^m^C and %^u^C^u^C increased, while the %^m^C^u^C decreased in current smokers (*p* = 0.002, 0.015, and <0.0001, respectively). Additionally, the lower %^m^C^u^C still persisted in persons who had stopped smoking for over 1 year (*p* = 0.001). The %^m^C^u^C also decreased in the higher pack-year smokers (*p* = 0.028). Smoking possibly altered ^m^C^u^C to ^m^C^m^C and ^u^C^u^C forms, and changes ^u^C^m^C to ^u^C^u^C forms. In conclusion, smoking changes methylation levels of partial methylated LINE-1s and increased the number of hypo- and hypermethylated loci. These hypomethylated LINE-1s may possess carcinogenesis potential. Moreover, LINE-1 methylation patterns may be useful for monitoring oral carcinogenesis in smokers.

## Introduction

Tobacco smoking is a predisposing factor of many malignancies [1]–[4]. The risk of upper aerodigestive cancers increases with the higher pack-years cigarette smoking [3], [5], [6]. However, this risk decreases after discontinuation of smoking and reverts to the non-smoker risk level if smoking is ceased for more than 15 years [3], [6]. Additionally, smoking increases the number of keratinised cells in the epithelium of the tongue and hard palate [7]. This effect varied in different regions, depending on the extent of direct exposure to smoke [8]. Interestingly, oral mucosal lesions resolved after cessation of smoking for a period of time [9], [10].

Mutations, promoter methylation and global hypomethylation are three crucial DNA modification events that lead to cancer development [11]–[13]. Smoking promotes mutations and alterations of gene promoter methylation [11], [14], [15]. Moreover, the evidence suggesting the association between the degree of global hypomethylation and smoking history of head and neck squamous cell carcinoma (HNSCC) patients was shown [16]. Long interspersed element-1s (LINE-1s) are repetitive transposable elements which are widely distributed in the genome [17]. There are 500,000 copies of LINE-1 in the human genome [18]. More than 10,000 LINE-1s contain a 5′UTR [19]. Most LINE-1 methylation studies, including this one, evaluated methylation at 5′UTR. The reduction of methylation levels of LINE-1, which reflects global hypomethylation, in various types of cancers had been extensively studied [20]. In most cancers, LINE-1 methylation levels diminish early and progressively which correlate significantly with tumour phenotype, including tumour progression and prognosis [12], [16], [21]–[26]. The hypomethylation of LINE-1 significantly increases the risk for HNSCC [27]. On the other hand, events associating LINE-1 hypermethylation with carcinogenesis have also been found in malignant peripheral nerve sheath tumor, myelodysplastic syndrome and partial hydatidiform moles [28]–[30].

In blood samples of HNSCC patients, LINE-1 methylation levels slightly increased with higher pack-years of smoking [27]. The effects of smoking on LINE-1 methylation levels in non-cancerous cells have also been reported. No changes were observed in blood cells or in the colonic epithelium of smokers *in vivo* [31]–[33]. However, an *in vitro* study revealed minimal reduction of LINE-1 methylation levels in the respiratory epithelium under high dosage cigarette smoke condensate treatment [34]. The oral mucosa is directly exposed to tobacco smoke and its chemical agents. Therefore, it is interesting to clarify whether this epigenetic change occurs before malignant transformation.

Currently, most LINE-1 methylation studies have measured the genome-wide methylation levels of LINE-1s. However, methylation of LINE-1s can be influenced by multiple mechanisms. The measurement of the methylation level alone may not be able to detect LINE-1 methylation changes in certain events, even if such changes can promote cancer development. In normal cells, some functions of LINE-1 methylation are to maintain genomic integrity and regulate gene expression in *cis* [20], [35]–[37]. Consequently, genomic instability and repression of gene expression can be observed on chromosomes in which LINE-1s are hypomethylated. Therefore, in theory, certain conditions that stochastically alter LINE-1 methylation levels will promote carcinogenesis on chromosomes with LINE-1 hypomethylation, but these hypomethylated LINE-1s may be undetectable because the methylation levels are counterbalanced by other hypermethylated LINE-1 loci. Locus-specific mechanisms causing variations in methylation levels among LINE-1s in different loci has also been reported [38].

**Figure 1:**
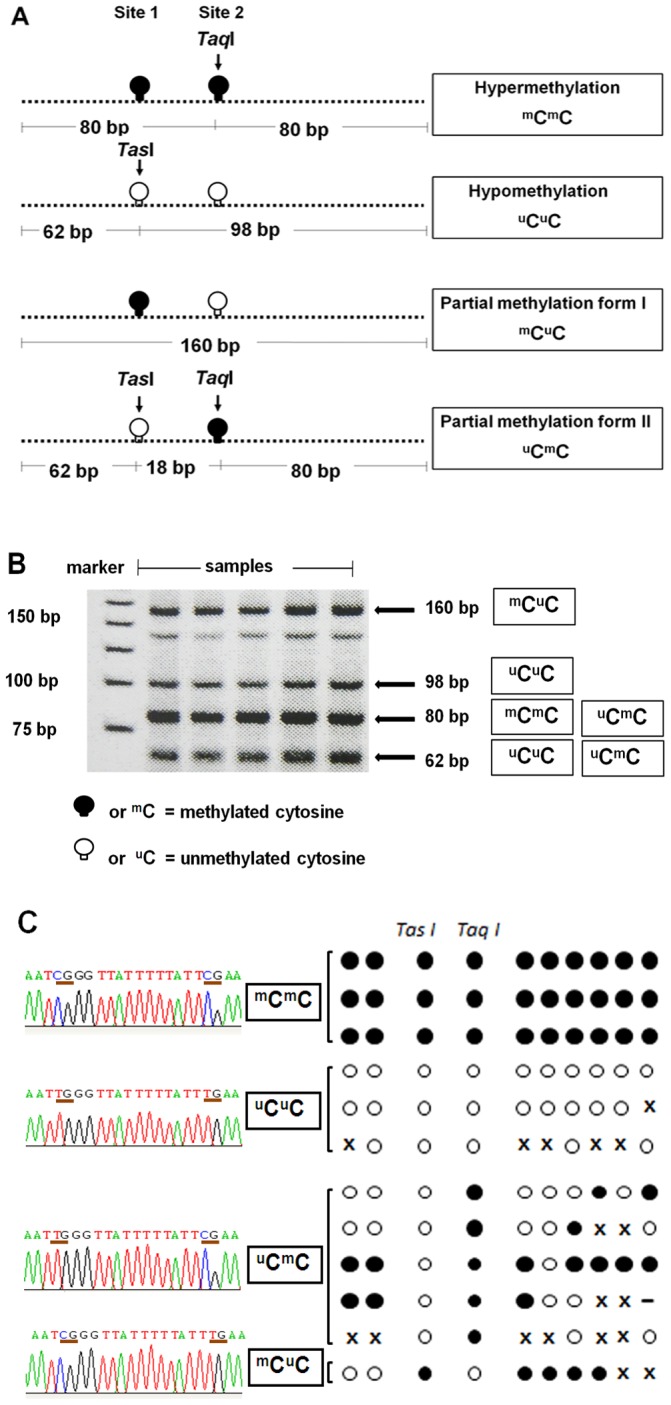
Methylation patterns of COBRALINE-1. (A) The LINE-1 amplicons were 160 bp and had 2 CpG dinucleotides. Four patterns of methylated CpGs were detected, including hypermethylation (^m^C^m^C), hypomethylation (^u^C^u^C), and two forms of partial methylation (^m^C^u^C and ^u^C^m^C). The *Tas*I enzyme targets unmethylated cytosine site 1, and *Taq*I targets methylated cytosine site 2. (B) After restriction digestion with *Tas*I and *Taq*I, four sizes of products (160, 98, 80, and 62 bp) were identified, depending on the methylation status of both CpG loci. (C) Examples of bisulfite sequencing. Left side represents sequences of the two CpG dinucleotides at *Tas*I and *Taq*I cut site whereas the right side represents the CpG dinucleotides in the amplified PCR products. Each circle exemplifies the methylation status of each selected clone. Black and white circles are methylated and unmethylated CpG dinucleotides, respectively. x is mutated sequence and **–** is deleted sequence.

Recently, a wide range of approaches to obtain quantitative information of genomic DNA methylation have been developed [39]. Most standard techniques measure several CpGs in each LINE-1. Pyrosequencing often measures 4 CpG dinucleotides [40], whereas combined bisulphite restriction analysis (COBRA) polymerase chain reaction often measures 2 CpGs [12]. Compared to previously reported LINE-1 sequences [38], the methylation state of 2 of the CpG dinucleotides detected by COBRALINE-1 correlated directly with other CpG dinucleotides on 5′LINE-1s. COBRA classifies LINE-1alleles into four groups depending on the methylation status of 2 CpG dinucleotides on each strand from 5′ to 3′ detected by COBRALINE-1. The first class contains 2 unmethylated CpGs (^u^C^u^C) and the second class contains 2 methylated CpGs (^m^C^m^C), representing hypomethylated and hypermethylated LINE-1 loci, respectively. The third and fourth classes are partially methylated LINE-1s including 5′methylated with 3′unmethylated CpGs (^m^C^u^C) and 5′ unmethylated with 3′methylated CpGs (^u^C^m^C) (Figure 1A and 1B). Recently, our group found that %^u^C^u^C is more effective in determining cancer risk than overall methylation levels [41]–[43].

However, studies that evaluated the association between smoking and repetitive sequence methylation changes *in vivo* have not yet been conclusive. Herein, we evaluated the possibility that smoking may promote cancer development via genomic hypomethylation by evaluating the human LINE-1 methylation pattern found in the oral mucosa of smokers.

## Results

### Methylation Status of LINE-1 Methylation Patterns

Twenty CpG dinucleotides of COBRALINE-1 clones were analysed and compared with ^m^C^m^C, ^u^C^u^C, ^m^C^u^C and ^u^C^m^C patterns. Examples of bisulphite DNA sequencing were demonstrated (Figure 1C). Most of the CpG dinucleotides of ^m^C^m^C were methylated. On the contrary, ^u^C^u^C were enriched in unmethylated CpGs. Finally, all alleles of ^m^C^u^C and ^u^C^m^C were partially methylated (Figure 1C).

### The Percentage of Each LINE-1 Methylation Pattern by Gender, Age and Alcohol Consumption

Oral rinses were collected from 60 non-smoker (NS) volunteers (35 males and 25 females) and 96 current smoker (CS) volunteers (80 males and 16 females) (Table 1). The imaging of the digestion products was used to generate %methylation value of each pattern. We evaluated if sex and age influenced LINE-1 methylation patterns. No significant difference in the percentage of LINE-1 products was detected between the males and females (Table S1). Furthermore, no correlation between each methylation pattern and age was found except a borderline increase in LINE-1 hypomethylation by age of CS group (*p* = 0.047, Table S2).

**Table 1:** Demographic and behavioral characteristics of the subjects.

		Non-smokers	Current smokers	Former smokers
**Total Subjects**		60	96	17
**Gender**	Male	35 (58.33%)	80 (83.33%)	15 (88.24%)
	Female	25 (41.67%)	16 (16.67%)	2 (11.76%)
**Age**	Mean ± SD	44.63±14.19	41.60±4.60	46.59±16.39
**History of alcohol drinking**	Currently drink	36 (60%)	86 (89.58%)	14 (82.35%)
	Previously drink	-	-	3 (17.65%)
	Never drink	24 (40%)	10 (10.42%)	-
**History of betel chewing**	Currently chew	-	-	-
	Previously chew	-	-	-
	Never chew	-	-	-

Most of the CS subjects in this study were also alcohol drinkers (Table 1). We found that in NS group, only %^m^C^u^C was significantly lower in the current drinker than in the never drink (*p* = 0.006). For CS group, only overall methylation level was higher in current drinker (*p* = 0.005, Table 2). However, the association between smoking and alcohol consumption and its contribution to malignant potency has not been completely elucidated. Therefore, before analysing the possible impact of smoking, we assessed the interaction between alcohol and smoking on every LINE-1 methylation patterns by using two-way ANOVA. No interactions between alcohol and smoking consumption were found for any of the patterns (*p*>0.05). Accordingly, the smoking factor could be independently studied, while controlling for alcohol consumption. The percentages of all the patterns are presented in Table S1.

**Table 2:** The percentages of LINE-1 products between never drinks and current drinkers.

	Non-smokers			Current smokers		
	Never drink	Currently drink	*p*-value^a^	Never drink	Currently drink	*p*-value^b^
**Number of subjects**	24	36		10	86	
**% ^m^C (mean ± SD)**	41.82±1.99	41.56±2.99	0.71	40.66±1.00	42.23±2.65	<0.01
**% ^m^C^m^C (mean ± SD)**	15.89±2.87	14.81±5.83	0.52	15.61±4.64	17.87±4.74	0.23
**%^u^C^u^C (mean ± SD)**	32.24±2.91	31.70±2.78	0.54	34.29±5.98	33.41±3.49	0.71
**% ^m^C^u^C (mean ± SD)**	28.41±2.69	25.82±3.72	<0.01	24.28±4.89	24.11±3.56	0.91
**% ^u^C^m^C (mean ± SD)**	23.47±4.34	27.67±8.81	0.11	25.81±9.20	24.61±7.71	0.70
**% ^m^C^u^C+^u^C^m^C (mean ± SD)**	51.88±4.19	53.49±6.91	0.29	50.10±10.51	48.72±6.42	0.61

a *t*-test was used to compare the percentage of LINE-1 products between never drink and currently drink of non-smokers.

b *t*-test was used to compare the percentage of LINE-1 products between never drink and currently drink of current smokers.

### The Percentages of Loci of Each LINE-1 Methylation Pattern in NS and CS were Different

**Figure 2:**
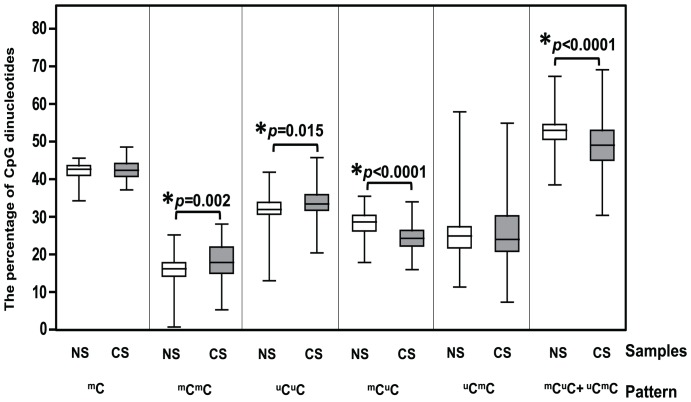
Percentage of each methylated CpG pattern in NS and CS. ^m^C represents the overall methylation level of the amplified LINE-1s. ^m^C^m^C and ^u^C^u^C represent hypermethylated and hypomethylated CpG of the amplified region, respectively. ^m^C^u^C and ^u^C^m^C represent two forms of partial methylation. ^m^C^u^C+^u^C^m^C is the sum of partially methylated loci of both forms. The horizontal line within each box indicates the mean of the percentage. Stars indicate statistical significance at *p*<0.05. The results demonstrated that the CS had a significantly higher %^m^C^m^C and %^u^C^u^C and a lower %^m^C^u^C and %^m^C^u^C+%^u^C^m^C than the NS.

Here we compared LINE-1 methylation between CS and NS. The CS had significantly higher %^m^C^m^C and %^u^C^u^C and lower %^m^C^u^C and %^m^C^u^C+^u^C^m^C than the NS (*p* = 0.002, 0.015, <0.0001, and <0.0001, respectively). However, no significant difference was found in overall methylation (%^m^C) and %^u^C^m^C (*p* = 0.33 and 0.84, respectively, Figure 2 and Table S1).

**Figure 3:**
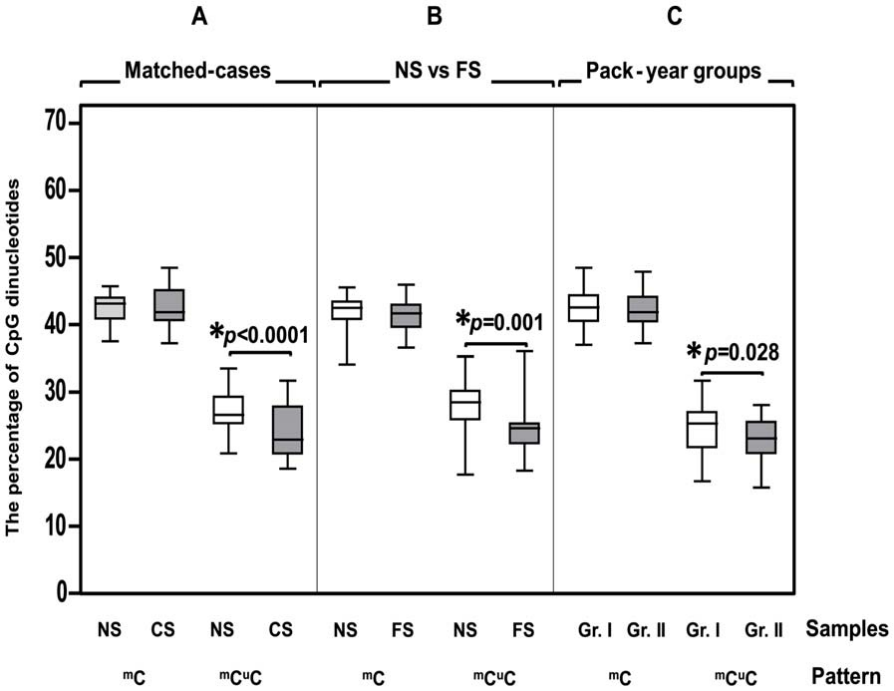
%^m^C^u^C in the matched cases, FS, and pack-year groups. ^m^C^u^C represented %^m^C^u^C. The percentage of overall LINE-1 methylation level was depicted as ^m^C. (A) To account for potential confounding factors, we matched the NS to the CS based on age, gender and alcohol drinking. The CS showed a significantly lower %^m^C^u^C. (B) The FS also had a significantly lower %^m^C^u^C. (C) %^m^C^u^C was significantly lower in the higher pack-year smoking (group II) than in lower pack-year group (group I).

To account for potential confounding by age, gender and alcohol consumption, the NS subjects were matched to the CS subjects based on age, gender and alcohol drinking behaviour, which produced 29 pairs. The same tendency of differences in the LINE-1 methylation patterns as those found in the total sample was found. However, only %^m^C^u^C resulted in a significant difference at *p*<0.0001 (Figure 3A and Table S1).

### Smoking Influenced the Methylation of LINE-1s

To study the persisting effect of smoking, an additional investigation was performed on 17 former smokers (FS),15 males and 2 females, who had quit smoking for no less than 1 year and who had no mucosal lesions. We found that the FS had a significant lower level of %^m^C^u^C than the NS (*p* = 0.001) while other patterns did not reveal any significant differences (Figure 3B and Table S1). This result implied the prolong effect of smoking on oral mucosa.

Based on the intensity of smoking, all of the smoking subjects were categorised into 2 groups based on the average pack-year (group I≤13.23and group II>13.23 pack-years). Only %^m^C^u^C was significantly different between the groups; %^m^C^u^C was significantly lower in group II, *p* = 0.028 (Figure 3C and Table S3). This result demonstrated the influence of smoking intensity on oral mucosa.

### The Possible Direction of LINE-1 Methylation Pattern Changes

**Figure 4:**
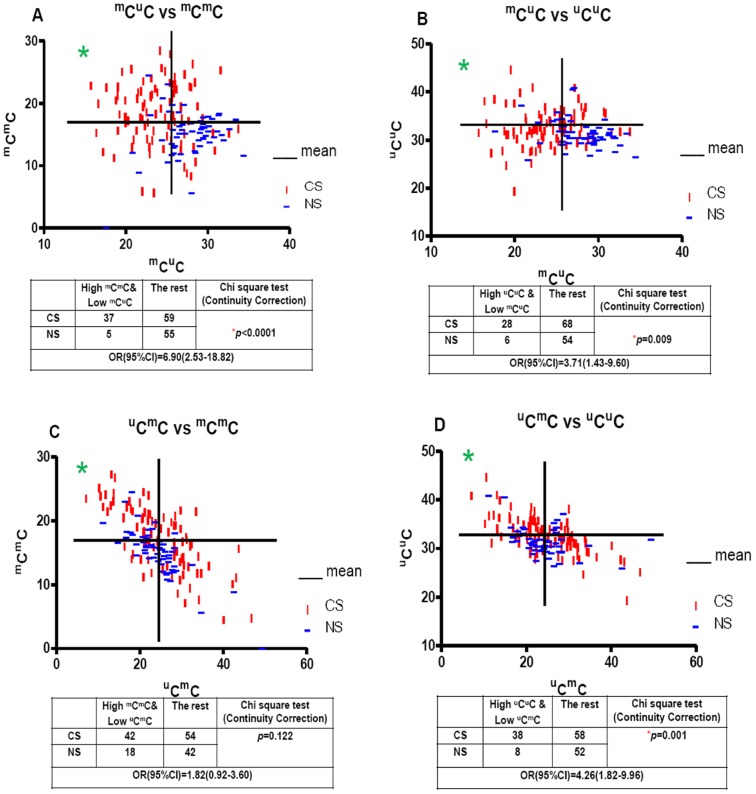
The possible direction of LINE-1 methylation pattern changes. ^m^C^u^C, ^u^C^m^C, ^m^C^m^C and ^u^C^u^C, represented %^m^C^u^C, %^u^C^m^C, %^m^C^m^C and %^u^C^u^C, respectively. The graphs were plotted for the percentages of either ^m^C^u^C or ^u^C^m^C on the X-axis and either ^m^C^m^C or ^u^C^u^C on the Y-axis. The vertical and horizontal lines indicate the mean percentages of each axis. The graph is divided into 4 quadrants. The numbers of NS and CS who fell in the upper left quadrant were counted and compared to the remainder of the group using the chi-squared test. The results are shown in the tables below the graphs.

Encouraged by the information that the change in ^m^C^u^C was opposite that of ^m^C^m^C and ^u^C^u^C, we checked whether the bivariate distributions of hetero-methylated/homo-methylated allelic proportions differed by smoking status. The number of CS who had a lower %^m^C^u^C concomitantly a higher %^m^C^m^C than the group means were counted and compared with the remainder of the group, also those of NS using the chi-squared test. We found that the odds ratio (OR) was 6.90 and the 95% confidential interval (CI) was 2.53–18.82 with *p*<0.0001 (Figure 4A). We performed the same test for the proportion of low ^m^C^u^C/high ^u^C^u^C, the OR and 95% CI were 3.71 and 1.43–9.60, respectively with *p* = 0.009 (Figure 4B). These results implied that a reduction of ^m^C^u^C in the CS was associated with an increase in ^m^C^m^C or ^u^C^u^C. Moreover, to clarify the possibility that the reduction of ^u^C^m^C is related to the increase in ^m^C^m^C or ^u^C^u^C in CS, the same analysis was performed in the group with low ^u^C^m^C/high either ^m^C^m^C or ^u^C^u^C. Though elevated in CS, the proportion of low ^u^C^m^C/high ^m^C^m^C subjects was not statistically different between CS and NS individuals (OR = 1.82, 95% CI = 0.92–3.60, *p* = 0.122, Figure 4C), while CS had a higher proportion of low ^u^C^m^C/high ^u^C^u^C relative to NS (OR = 4.26, 95% CI = 1.82–9.96, *p* = 0.001, Figure 4D). This result suggested that the reduction of ^u^C^m^C might associate with the increase in ^u^C^u^C but not ^m^C^m^C.

## Discussion

This study confirms that COBRALINE-1 does not only analyse the overall methylation level, but it is also able to show the methylation pattern of LINE-1s.We found that ^u^C^u^C and ^m^C^m^C represent the hypomethylation and hypermethylation of LINE-1, respectively. Moreover, ^m^C^u^C and ^u^C^m^C also represent partial methylation.

Although we did not find differences in LINE-1 methylation level by age and gender, the influences by these two factors in blood cells were demonstrated [44]–[46]. However, the degrees of differences by ages and gender were minimal when comparing with differences by environmental insults or diseases [12], [47], [48]. Therefore, the differences by age and gender could be found only when large cohorts were studied. Here, although the relationship of age and LINE-1 methylation pattern of oral mucosa was not found in this study, further investigation should be performed in larger sample size and in a variety of age.

Similar to blood cells and colonic epithelium [31]–[33], cigarette smoke does not change overall LINE-1 methylation level of oral mucosa. However, there are alterations in patterns of LINE-1 methylation that we found both ^u^C^u^C and ^m^C^m^C loci increased. The unchanged overall methylation level can be explained by the fact that the LINE-1 methylation level is a sum of the methylation from all the LINE-1s. Therefore, the increases in both the ^u^C^u^C and ^m^C^m^C loci counterbalance each other, neutralising their effect on the overall LINE-1 methylation levels. This evidence supported by our previous study, which revealed that the overall LINE-1 methylation level measurement was not sufficiently sensitive or accurate to determine the LINE-1 methylation changes in pathological conditions [20]. We recommend re-evaluating LINE-1 methylation pattern to most of the previous studies reported unaltered overall methylation level.

Contrary to the reduction of genome-wide methylation levels caused by some chemical agents [49], [50], smoking could promote both an increase and decrease methylation in certain LINE-1s. Interestingly, while smoking-induced ^u^C^u^C, hypomethylated LINE-1 loci, possibly originated from both forms of partially methylated LINE-1(^m^C^u^C and ^u^C^m^C), the ^m^C^m^C might derive from only one partially methylated form, ^m^C^u^C. These observations suggest that the mechanisms which increase or decrease methylation are different. The methylation loss mechanism seems to be a generalised process that affects many LINE-1s regardless of the original methylation patterns and is similar to the global hypomethylation found in cancer [20], [24], [38]. Accordingly, cancer and smoking may reduce genome-wide methylation by the same mechanism.

**Figure 5:**
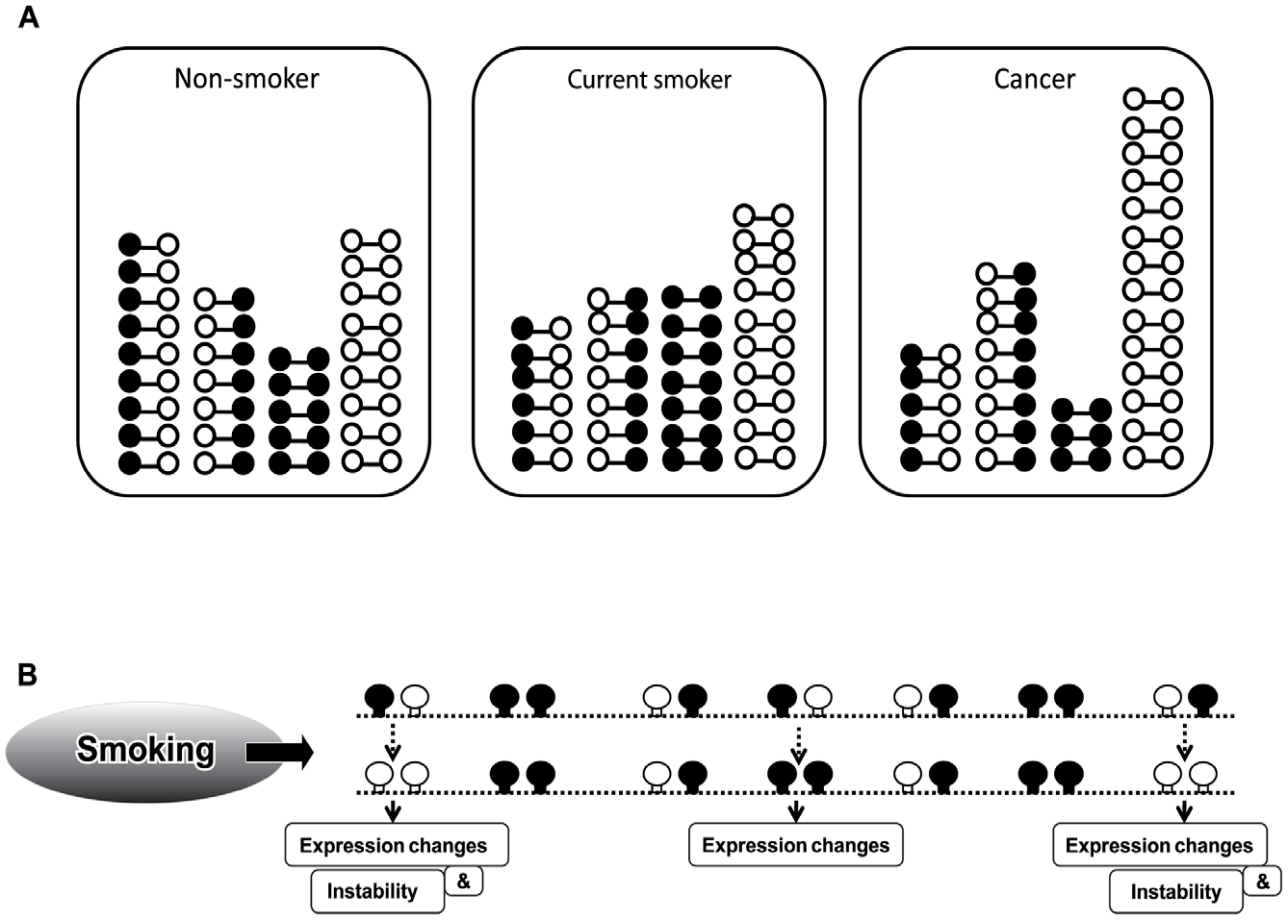
The influence of smoking on the epigenetic progression of multistep carcinogenesis. (A) Models of LINE-1 methylation patterns in oral mucosal cells of a NS, the oral mucosal cells of a CS and in cancer cells (HeLa) are shown. Although the overall methylation level did not change in the CS, some alterations in the methylation patterns were detected. While the numbers of ^m^C^m^C and ^u^C^u^C were increased, only one form of partial methylation, ^m^C^u^C, was decreased. Moreover, the addition of ^m^C^m^C and ^u^C^u^C correlated with the depletion of ^m^C^u^C. In contrast, a reduction in the overall methylation level was found in cancer cells. The numbers of ^m^C^m^C and ^m^C^u^C were significantly decreased, while the numbers of ^u^C^u^C were significantly increased. The numbers of ^u^C^m^C were slightly increased. (B) The smoking-induced hypomethylated loci could be derived from both classes of the partial methylation patterns and could result in genome instability and gene expression changes. However, the smoking-induced hypermethylated loci were from ^m^C^u^C only and, consequently, effected gene expression.

Even though the methylation differences between smokers and non-smokers are just a few percentage points difference, the alteration may play a role in carcinogenesis. Global hypomethylation can cause cancer by promoting genomic instability and by altering gene expression in *cis*, on that same chromosome [20]. There are evidences suggesting that DNA methylation maintains genomic integrity in *cis.* First, a close correlation between the site of the chromosome translocation and the loss of the methylation of satellite DNA has been reported [51], [52]. Recently, we found that the repair of the replication of independent DNA double-strand breaks occurring within hypomethylated regions was more error prone [36]. For gene expression, we reported the repression of mRNA production by hypomethylated intragenic LINE-1s [37]. Therefore, the increasing number of hypomethylated LINE-1 loci, which implies the hypomethylation, induced by smoking should promote cancer at certain loci in *cis* (Figure 5).

Although no interaction between alcohol consumption and cigarette smoking was found in LINE-1 methylation, similar to a report in colon [53], the result revealed some significant change in alcohol drinkers. When there is no effect of smoking, the oral mucosa of drinkers has lower %^m^C^u^C than never drink persons. For the persons who drink and smoke, the overall methylation level of LINE-1s increases while the patterns are not affected. This information also needs further investigation of the result of chemical exposure to oral mucosa.

In conclusion, smoking alters LINE-1 methylation by increasing or decreasing methylation of certain loci. The mechanisms causing LINE-1 loss or gain methylation are different. The alteration can persist after stop smoking. Moreover, the higher intensity of smoking results in the higher alteration degree. Further exploration of methylation pattern changes of other intersperse repetitive sequences and gene promoters whether they are related to other smoking-associated malignancies, as well as other carcinogens, is necessary. Finally, a better understanding of the causes and mechanisms of genome-wide methylation changes will be crucial for cancer prevention.

## Materials and Methods

### Ethics Statement

The Ethics Committee of the Faculty of Dentistry, Chulalongkorn University approved this study (approval number: 7/2010). Written informed consent was obtained from every participant to the study.

### Oral Mucosal Cell Collection

The patients who received dental treatment in the Faculty of Dentistry during December 2010 to May 2011 were voluntarily enrolled. After oral examination and history taking, the patients who had no oral mucosal lesion and no history of malignancy of any organs were recruited in this study. The number and duration of cigarette smoking were recorded if they had the smoking history. The alcohol consumption was also recorded as never or ever drinks. CS included the patients who had still smoked at the time of interview. NS included the patients who had never smoked. FS, the patients who quit smoking longer than 1 year were also included in this study. Oral epithelia were collected from oral rinses. Ten millilitres of sterile 0.9% normal saline solution was gargled for 15 seconds. This solution was kept in a sterile tube and stored at 4°C until the DNA extraction process.

### Genomic DNA Extraction

After oral rinses were centrifuged at 4°C, 2500 g for 15 minutes, the supernatant was discarded. The cell pellets were washed twice in sterile PBS. One millilitre of the DNA extraction buffer with 10% SDS and proteinase K (0.5 mg/ml) was added to the cell pellets. The mixtures were then incubated at 50°C for two nights. A phenol-chloroform extraction was used to purify and desalt the digested cell pellets. After centrifuging at 4°C, 14000 g for 15 minutes, 10 M ammonium acetate and cold absolute ethanol were added to the upper aqueous phase for DNA precipitation. The precipitated DNA was washed with 70% ethanol. The air-dried DNA was then resuspended in Tris-EDTA-treated water.

### COBRALINE-1

COBRA for LINE-1 was performed as previously described, the 5′UTR of LINE-1.2 (L1Hs) sequence from NCBI Accession Number M80343 was used [12]. In brief, the DNA samples were converted by a bisulphite reaction such that unmethylated cytosine (^u^C) would be converted to uracil (U), whereas methylated cytosine (^m^C) would remain as cytosine. One microlitre of bisulphite DNA was then subjected to 35 cycles of PCR, at a 50°C annealing temperature using the following primer sets: LINE-1-F (5′-CCGTAAGGGGTTAGGGAGTTTTT-3′) and LINE-1-R (5′-RTAAAACCCTCCRAACCAAATATAAA-3′). The LINE-1 amplicons (160 bp) were digested with 2 U of *Taq*I and 2 U of *Tas*I in NEB3 buffer (New England Biolabs, Ontario, Canada) at 65°C overnight. The products were identified by polyacrylamide gel electrophoresis (8% non-denaturing) and stained with SYBR green nucleic acid stain (Sigma-Aldrich, St. Louis, Missouri). Distilled water was used as a negative control. The same preparation of DNA from 3 cell lines, HeLa (cervical cancer), Daudi (Human Burkitt’s lymphoma), and Jurkat (acute T cell leukemia) (ATCC, Manassas, VA, USA) were used as positive controls in all experiments and for inter-assay variation adjustment [41].

### COBRALINE-1 Product Analysis and Bisulphite DNA Sequencing

Here, we classified LINE-1s into four groups depending on the methylation status of 2 CpG dinucleotides on each strand from 5′ to 3′ detected by COBRALINE-1 as described previously [42]. These COBRA-detected LINE-1s were categorised into the following four classes: 2 unmethylatedCpGs (^u^C^u^C), 2 methylated CpGs (^m^C^m^C), 5′methylated and 3′unmethylated CpGs (^m^C^u^C), or 5′unmethylated and 3′methylated CpGs (^u^C^m^C) (Figure 1A). LINE-1 methylation levels and the percentage of loci of each class were calculated from COBRALINE-1 digested products. Intensities of COBRALINE-1 bands were measured by a phosphoimager using ImageQuant Software (Molecular Dynamics, GE Healthcare, Slough, UK). After enzymatic digestion, the COBRALINE-1 amplicons were separated into 5 DNA strands depending on their length, 160, 98, 80, 62, and 18 bp (Figure 1B). The 18 bp band was not used in the following calculation. The 160 bp band contains 2 CpGs, in which the 5′CpG is methylated and the other 3′CpG is unmethylated. The 98 bp band contains 2 unmethylated CpGs. The 80 bp and 62 bp bands each contain 1 methylated and 1 unmethylated CpG. The CpGs of the 160 bp and 98 bp bands were derived from ^m^C^u^C and ^u^C^u^C, respectively. The CpGs of the 80 bp band were derived from 3′methylated CpGs of ^m^C^m^C and ^u^C^m^C, respectively, while the CpGs of the 62 bp band were derived from 5′unmethylated CpGs of ^u^C^u^C and ^u^C^m^C (Figure 1). To normalise each band to represent the total number of CpG dinucleotides present, the intensity of each band was divided by the number of base pairs of double stranded DNA as follows: %160/160 = A, %98/94 = B, %80/78 = C, and %62/62 = D. Then, the LINE-1 methylation levels were computed with the following formula: percentage of overall LINE-1 methylation level (%^m^C) = 100×(C+A)/(C+A+A+B+D), percentage number of ^m^C^u^C loci (%^m^C^u^C) = 100×(A)/(((C-D+B)/2)+A+D), %^u^C^m^C = 100×(D-B)/((C-D+B)/2)+A+D, %^u^C^u^C = 100×B/(((C-D+B)/2)+A+D), and %^m^C^m^C = 100×((C-D+B)/2)/(((C-D+B)/2)+A+D).

To analyse methylation status of each LINE-1 methylation pattern, COBRALINE-1 PCR products were cloned into the pGEM-T easy vector (Promega, Santhan, UK) and sequenced.

### Statistical Analysis

Statistical analyses were performed using SPSS version 17.0 (SPSS Inc., Chicago, IL). All *p*-values less than 0.05 were considered significant. All the variables were normally distributed (Kolmogorov-Smirnov Test). We used a two-way analysis of variance (ANOVA) to determine the effects of two factors, alcohol and smoking, on the methylation levels of LINE-1. The independent sample *t*-test was performed to compare each LINE-1 methylation pattern between males and females, NS and CS, NS and FS, the 2 pack-year groups also never drinks and current drinker. In addition, the paired *t*-test was used for a matched-case analysis. The relationship of age and LINE-1 methylation was investigated with Pearson’s correlation. The chi-squared test and OR were used to test the association among LINE-1 methylation variables in CS and NS.

## Supporting Information

Table S1 **The percentage of LINE-1 products in smokers and non-smokers.**
(DOC)

Table S2 **Relationship of the percentage of LINE-1 products with age status.**
(DOC)

Table S3 **Demographic characteristics and the percentage of LINE-1 products in pack-year smoking groups.**
(DOC)
