# Supplementary material for: Patterns and Possible Roles of LINE-1 Methylation Changes in Smoke-Exposed Epithelia
Source: PLoS One. 2012 Sep 18;7(9):e45292. doi: 10.1371/journal.pone.0045292 (PMC3445447; doi:10.1371/journal.pone.0045292)
Supplement: Table S1 — The percentage of LINE-1 products in smokers and non-smokers. (DOC) [file pone.0045292.s001.doc]

**Table S1. The percentage of LINE-1 products in smokers and non-smokers**.

|  |  | **All cases** | | | | | | | | | | | | | | | | | | | | | | | | | | | | | | | | | | **Matched cases** | | | | | | | | | | | | |
| --- | --- | --- | --- | --- | --- | --- | --- | --- | --- | --- | --- | --- | --- | --- | --- | --- | --- | --- | --- | --- | --- | --- | --- | --- | --- | --- | --- | --- | --- | --- | --- | --- | --- | --- | --- | --- | --- | --- | --- | --- | --- | --- | --- | --- | --- | --- | --- | --- |
|  | **Non-smokers** | | | | | | |  | | **Current smokers** | | | | | | | | | |  | | **Former smokers** | | | | | | | | | | |  | | **Non-smokers** | | | | |  | | **Current smokers** | | | | | | |
|  | **Total** | | **Male** | **Female** | | ***p*-value^a^** | |  | | | | **Total** | **Male** | | **Female** | | ***p*-value^a^** | |  | | | **Total** | **Male** | **Female** | | ***p*-value^a^** | | ***p*-value^b^ *p*-value^c^** | | | |  | | **Total** | | | **Male** | **Female** | ***p*-value^a^** | |  | | **Total** | **Male** | **Female** | ***p*-value^a^** | | ***p*-value^d^** |
| **Number of subjects** | **60 (100%)** | | **35 (58.37%)** | | **25 (41.67%)** | |  | |  | | **96 (100%)** | | | **80 (83.33%)** | | **16 (16.67%)** | |  |  | | **17 (100%)** | | **15 (88.24%)** | | **2 (11.76%)** | |  | |  |  |  | | | **29** | | | **14** | **15** |  | |  | | **29** | **14** | **15** |  |  | |
| **Age (year ± SD)** | **44.63 ± 14.19** | | **44.29 ± 17.66** | | **44.74 ± 3.18** | |  | |  | | **41.60 ± 4.60** | | | **40.78 ± 16.16** | | **42.29 ± 7.68** | |  |  | | **46.59±16.39** | | **48.88 ± 15.91** | | **30.00 ± 2.83** | |  | |  |  |  | | | **45.41 ± 5.33** | | | **44.29 ± 17.66** | **46.47 ± 3.34** |  | |  | | **44.59 ± 4.13** | **44.07 ± 6.25** | **45.07 ± 12.39** |  |  | |
| **% ^m^C (mean ± SD)** | **41.76 ± 2.22** | | **42.55 ± 1.82** | | **41.51 ± 2.32** | | **0.13** | |  | | **42.16 ± 2.62** | | | **42.34 ± 2.62** | | **41.29 ± 2.49** | | **0.14** |  | | **41.16 ± 2.38** | | **41.01 ± 2.44** | | **42.22 ± 2.28** | | **0.52** | | **0.33** | **0.34** |  | | | **42.28 ± 2.23** | | | **42.55 ± 1.82** | **42.02 ± 2.59** | **0.53** | |  | | **42.32 ± 3.12** | **43.44 ± 2.79** | **41.29 ± 3.14** | **0.06** | **0.95** | |
| **% ^m^C^m^C (mean ± SD)** | **15.63 ± 3.72** | | **16.94 ± 4.03** | | **15.22 ± 3.61** | | **0.13** | |  | | **17.82 ± 4.79** | | | **18.21 ± 4.68** | | **15.93 ± 5.03** | | **0.82** |  | | **15.47 ± 4.61** | | **15.47 ± 4.95** | | **15.49 ± 0.38** | | **0.10** | | **<0.01** | **0.89** |  | | | **17.38 ± 4.54** | | | **16.94 ± 4.03** | **17.78 ± 5.07** | **0.63** | |  | | **17.49 ± 5.35** | **18.30 ± 4.69** | **15.81 ± 5.54** | **0.08** | **0.92** | |
| **%^u^C^u^C (mean ± SD)** | **32.11 ± 2.84** | | **31.84 ± 2.32** | | **32.20 ± 3.04** | | **0.69** | |  | | **33.50 ± 3.74** | | | **33.53 ± 3.83** | | **33.35 ± 3.38** | | **0.85** |  | | **33.16 ± 1.97** | | **33.46 ± 1.35** | | **31.07 ± 4.95** | | **0.62** | | **0.02** | **0.17** |  | | | **32.82 ± 3.40** | | | **31.84 ± 2.32** | **33.74 ± 4.03** | **0.14** | |  | | **32.84 ± 4.31** | **32.43 ± 4.88** | **33.23 ± 3.84** | **0.62** | **0.99** | |
| **% ^m^C^u^C (mean ± SD)** | **27.85 ± 3.13** | | **26.73 ± 2.13** | | **28.12 ± 3.33** | | **0.15** | |  | | **24.03 ± 3.64** | | | **23.83 ± 3.61** | | **25.03 ± 3.74** | | **0.23** |  | | **24.51 ± 3.97** | | **24.72 ± 4.14** | | **23.07 ± 2.89** | | **0.60** | | **<0.01** | **<0.01** |  | | | **26.97 ± 2.93** | | | **26.73 ± 2.13** | **27.19 ± 3.59** | **0.68** | |  | | **23.89 ± 3.77** | **22.59 ± 3.55** | **25.09 ± 3.66** | **0.07** | **<0.01** | |
| **% ^u^C^m^C (mean ± SD)** | **24.41 ± 5.88** | | **24.49 ± 4.50** | | **24.46 ± 6.33** | | **0.99** | |  | | **24.64 ± 7.81** | | | **24.43 ± 8.02** | | **25.69 ± 6.80** | | **0.56** |  | | **26.85 ± 5.67** | | **26.35 ± 5.46** | | **30.37 ± 8.22** | | **0.37** | | **0.84** | **0.14** |  | | | **22.83 ± 5.92** | | | **24.48 ± 4.50** | **21.29 ± 6.78** | **0.15** | |  | | **25.78 ± 7.99** | **25.68 ± 9.18** | **25.86 ± 7.04** | **0.96** | **0.08** | |
| **% ^m^C^u^C+^u^C^m^C (mean ± SD)** | **52.26 ± 4.90** | | **51.22 ± 5.48** | | **52.59 ± 4.79** | | **0.37** | |  | | **48.68 ± 6.82** | | | **48.26 ± 6.76** | | **50.72 ± 6.98** | | **0.19** |  | | **51.36 ± 5.25** | | **51.07 ± 5.37** | | **53.44 ± 5.33** | | **0.57** | | **<0.01** | **0.52** |  | | | **49.80 ± 6.66** | | | **51.22 ± 5.48** | **48.49 ± 7.55** | **0.28** | |  | | **49.66 ± 7.45** | **48.28 ± 7.77** | **50.96 ± 7.16** | **0.34** | **0.93** | |

^a^ *t*-test was used to compare the percentage of LINE-1 products between male and female in each group

^b^ *t*-test was used to compare the percentage of LINE-1 products between non-smokers and current smokers

^c^ *t*-test was used to compare the percentage of LINE-1 products between non-smokers and former smokers

^d^ *t*-test was used to compare the percentage of LINE-1 products between non-smokers and current smokers in matched-cases analysis.
