# Supplementary material for: Patterns and Possible Roles of LINE-1 Methylation Changes in Smoke-Exposed Epithelia
Source: PLoS One. 2012 Sep 18;7(9):e45292. doi: 10.1371/journal.pone.0045292 (PMC3445447; doi:10.1371/journal.pone.0045292)
Supplement: Table S2 — Relationship of the percentage of LINE-1 products with age status. (DOC) [file pone.0045292.s002.doc]

**Table S2. Relationship of the percentage of LINE-1 products with age status**.

|  |  | Non-smokers | |  | Current smokers | |
| --- | --- | --- | --- | --- | --- | --- |
| Methylation patterns |  | Pearson correlation (r) | *p*-value^a^ |  | Pearson correlation (r) | *p*-value^b^ |
| % ^m^C |  | -0.089 | 0.50 |  | -0.204 | <0.05 |
| % ^m^C^m^C |  | -0.094 | 0.48 |  | -0.079 | 0.45 |
| %^u^C^u^C |  | 0.015 | 0.91 |  | 0.184 | 0.07 |
| % ^m^C^u^C) |  | 0.173 | 0.19 |  | 0.007 | 0.95 |
| % ^u^C^m^C |  | -0.039 | 0.77 |  | -0.43 | 0.68 |
| % ^m^C^u^C+^u^C^m^C |  | 0.063 | 0.64 |  | -0.46 | 0.66 |

^a^ Pearson correlation was used to identify correlation between LINE-1 methylation patterns

and age of non-smokers

^b^ Pearson correlation was used to identify correlation between LINE-1 methylation patterns and age of current smokers
