# Supplementary material for: Patterns and Possible Roles of LINE-1 Methylation Changes in Smoke-Exposed Epithelia
Source: PLoS One. 2012 Sep 18;7(9):e45292. doi: 10.1371/journal.pone.0045292 (PMC3445447; doi:10.1371/journal.pone.0045292)
Supplement: Table S3 — Demographic characteristics and the percentage of LINE-1 products in pack-year smoking groups. (DOC) [file pone.0045292.s003.doc]

**Table S3. Demographic characteristics and the percentage of LINE-1 products in pack-year smoking groups.**

|  | Pack-year smoking | |  |
| --- | --- | --- | --- |
|  | ≤13.23 (group I) | >13.23 (group II) | *p*-value^a^ |
| Number of subjects  Toatal= 96 (Current smokes) | 54 | 42 |  |
| Gender |  |  |  |
| Male | 44 | 36 |  |
| Female | 10 | 6 |  |
| Age (year ± SD) | 33.34 ± 10.26 | 52.31 ± 10.63 |  |
| % ^m^C (mean ± SD) | 42.32 ± 2.83 | 42.09 ± 2.44 | 0.69 |
| % ^m^C^m^C (mean ± SD) | 17.69 ± 5.10 | 18.39 ± 4.65 | 0.51 |
| %^u^C^u^C (mean ± SD) | 33.05 ± 4.00 | 34.22 ± 3.44 | 0.16 |
| % ^m^C^u^C (mean ± SD) | 24.30 ± 3.43 | 22.71 ± 3.05 | 0.03 |
| % ^u^C^m^C (mean ± SD) | 24.97 ± 8.42 | 24.69 ± 7.36 | 0.87 |
| % ^m^C^u^C+^u^C^m^C (mean ± SD) | 49.27 ± 7.22 | 47.39 ± 6.57 | 0.22 |

^a^ *t*-test was used to compare the percentage of LINE-1 products between pack-year smoking groups
